# Osteoblastoma arising from the orbital roof

**DOI:** 10.11604/pamj.2013.16.101.3513

**Published:** 2013-11-17

**Authors:** Zouheir Hafidi, Rajae Daoudi

**Affiliations:** 1 Université Mohammed V Souissi, Service d'Ophtalmologie A de l'hôpital des spécialités, Centre hospitalier universitaire, Rabat, Maroc

**Keywords:** Osteoblastoma, orbital roof, bone neoplasm, MRI

## Image in medicine

Osteoblastoma is a rare benign osteogenic bone neoplasm; it represents 1 to 3% of all bone tumors and usually seen in the second decade of life with a predilection in males. Classically it develops in the vertebral column. The skull is rarely involved and the orbital roof was reported to be less frequently involved. We report a new case of a benign osteoblastoma arising from the orbital roof with an intracranial and intra orbital extension. A 28-year-old woman presented with a recent history of rapidly progressive left exophthalmos, unilateral vision loss and headache. Her medical history was unremarkable. At examination there was a downward proptosis of the left eye with complete ophthalmoplegia. Visual acuity was 20/200 in the left eye with an increased intraocular pressure. Funduscopy revealed subtotal optic nerve cupping. On computed tomography there was a well sumscribed hyperdense mass arising from the horizontal part of the frontal bone with intraorbital and anterior cranial fossa extension. Magnetic Resonance Imaging showed a homogeneous extraconal mass with low signal intensity on T1-weighted imaging, there were an anterior displacement of the left eyeball which was compressed by the tumor and a superior displacement of the left frontal lobe. This was consistent with a frontal bone osteoblastoma. A complete surgical removal was decided. This was performed within a left-sided subfrontal approach with extradural exposure of the anterior skull base. Histopathological examination revealed a mature benign osteoblastoma. Postoperatively the patient resolved her proptosis. However the visual acuity remained unchanged. The main differential diagnosis of osteoblastoma is osteoid osteoma. Osteablastoma typically involves the axial skeleton and exhibits a high growth potential, while the osteoid osteoma occurs in the long bones and is much less aggressive. On computed tomography osteoblastoma presents typically as a dense, sclerotic, homogenous mass with well-defined margins and thin cortical margin. On MRI the tumor generally shows low-signal intensity on both T1 and T2 weighted studies without gadolinium enhancement. Regardless of tumor location a total resection should be attempted to avoid local recurrences; which is pretty difficult when there is a cranial base extension. Adjuvant radiotherapy for residual tumor may then be discussed.

**Figure 1:**
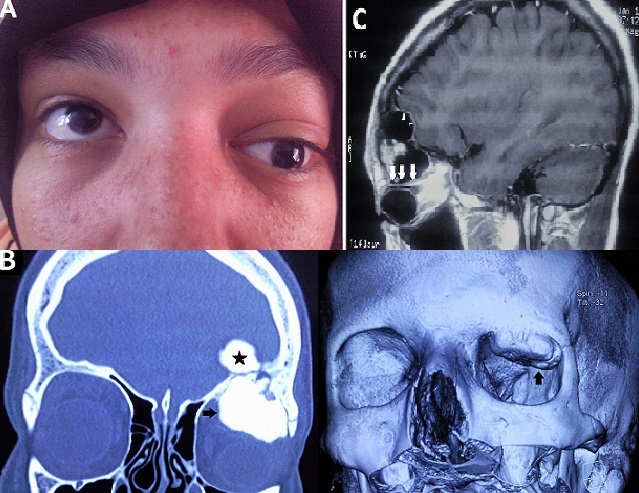
A): proptosis of the left eye with total ophthalmoplegia; B): coronal computed tomography (CT) and Three dimensional CT image of the cranium showing a right hyperdense intraorbital mass of the horizontal part of the frontal bone arising from its lateral side (black arrows) with an extension to the endocranium (star) C): Magnetic Resonance Imaging showing anterior displacement and compression of the left eyeball (white arrows) and the frontal lobe (white arrowheads)

